# Immunoinformatic Analysis Reveals Antigenic Heterogeneity of Epstein-Barr Virus Is Immune-Driven

**DOI:** 10.3389/fimmu.2021.796379

**Published:** 2021-12-16

**Authors:** Ana Cirac, Remy Poirey, Michael Dieckmeyer, Klaus Witter, Henri-Jacques Delecluse, Uta Behrends, Josef Mautner

**Affiliations:** ^1^ Children’s Hospital, School of Medicine, Technische Universität München, Munich, Germany; ^2^ German Centre for Infection Research (DZIF), partner site Munich, Munich, Germany; ^3^ German Cancer Research Center (DKFZ) Unit F100 and Institut National de la Santé et de la Recherche Médicale Unit U1074, Heidelberg, Germany; ^4^ Department of Diagnostic and Interventional Neuroradiology, Technische Universität München, Munich, Germany; ^5^ Laboratory of Immunogenetics, Ludwig-Maximilians-Universität, München, Germany; ^6^ Institute of Virology, Helmholtz Zentrum München, Munich, Germany

**Keywords:** Epstein-Barr virus, virus evolution, immunoinformatics, T-cell epitope, strain variants

## Abstract

Whole genome sequencing of Epstein-Barr virus (EBV) isolates from around the world has uncovered pervasive strain heterogeneity, but the forces driving strain diversification and the impact on immune recognition remained largely unknown. Using a data mining approach, we analyzed more than 300 T-cell epitopes in 168 published EBV strains. Polymorphisms were detected in approximately 65% of all CD8+ and 80% of all CD4+ T-cell epitopes and these numbers further increased when epitope flanking regions were included. Polymorphisms in CD8+ T-cell epitopes often involved MHC anchor residues and resulted in changes of the amino acid subgroup, suggesting that only a limited number of conserved T-cell epitopes may represent generic target antigens against different viral strains. Although considered the prototypic EBV strain, the rather low degree of overlap with most other viral strains implied that B95.8 may not represent the ideal reference strain for T-cell epitopes. Instead, a combinatorial library of consensus epitopes may provide better targets for diagnostic and therapeutic purposes when the infecting strain is unknown. Polymorphisms were significantly enriched in epitope versus non-epitope protein sequences, implicating immune selection in driving strain diversification. Remarkably, CD4+ T-cell epitopes in EBNA2, EBNA-LP, and the EBNA3 family appeared to be under negative selection pressure, hinting towards a beneficial role of immune responses against these latency type III antigens in virus biology. These findings validate this immunoinformatics approach for providing novel insight into immune targets and the intricate relationship of host defense and virus evolution that may also pertain to other pathogens.

## Introduction

The Epstein-Barr virus (EBV) establishes lifelong persistent infections in more than 90% of the human population by latently infecting B cells. Primary infection with EBV usually occurs early in life by parent-to-child oral transmission in an almost always asymptomatic fashion. Delayed primary infection in adolescence or adulthood may cause the syndrome of infectious mononucleosis (IM). Besides IM, EBV has been associated with a growing number of non-malignant and malignant diseases, such as chronic active EBV infection, hemophagocytic lymphohistiocytosis, post-transplant lymphoproliferative disease, NK/T-cell, Burkitt, and Hodgkin lymphoma, as well as nasopharyngeal and gastric carcinoma (1, 2). In addition, EBV infection has been linked to the etiology of several autoimmune diseases, e.g. multiple sclerosis (1, 3).

Based on the EBV nuclear antigen (EBNA) 2 and EBNA-3 gene sequences, EBV strains have been grouped into type 1 and type 2 (4–6). More recently, sequence analyses of a rapidly growing number of viral isolates from different healthy virus carriers and tumor specimens have identified numerous viral variants and complex variations in EBV strains within the 1/type 2 classification system (5, 7–10). Moreover, a comparative analysis of whole genome sequences identified a number of polymorphisms that were shared by tumor-derived strains but absent in viral isolates from healthy study participants (7). These findings suggest that some sequence variations may impact on viral phenotype and pathogenicity, a notion recently substantiated by *in vitro* studies (11–14).

In most virus carriers, EBV is contained as asymptomatic infection mainly by T cells recognizing various viral latent and lytic cycle antigens (15, 16). Consequently, patients with T-cell dysfunction are at increased risk of developing life-threatening EBV-associated lymphoproliferative disease and reconstitution of EBV-specific immunity in these patients by the adoptive transfer of virus-specific T-cells has been shown to be effective in preventing and treating such EBV-associated pathologies (17–21). However, response rates in apparently immunocompetent patients with EBV-associated malignancies are still unsatisfactory (19).

Owing to high viral titers that can be readily obtained from the supernatant of the producer cell line, the B95.8 EBV strain has become the prototypic type 1 strain and is commonly used for generating EBV-specific T-cell lines for clinical application (22). Whether immune responses elicited against B95.8 also protect against a rapidly growing number of heterogeneous field strains is still unknown. Amino acid sequence exchanges have been identified in several T-cell epitopes. Also, differences in T-cell recognition of target cells infected with different viral strains (23), or loaded with peptide epitope variants, have been described. In fact, the first EBV-derived CD8+ T-cell epitope that was molecularly defined was shown to differ between B95.8 and the prototypic type 2 strain AG876 in two amino acids (24). Among the CD8+ T-cell epitopes identified in subsequent studies, some proved to be conserved in different viral strains (25), while others were found to vary between virus types (26), or even within the same virus type (27–32). Depending on the epitope and the polymorphism, T-cell recognition was unaffected, diminished, or abolished (27–30, 32, 33).

While these investigations had focused on a limited number of CD8+ T-cell epitopes and diverse viral isolates, a recent study compared the majority of all published CD8+ and CD4+ EBV T-cell epitopes in the three viral strains B95.8, AG876, and M81 (34). Polymorphisms were detected in about half of all T-cell epitopes and the individual T-cell responses ignored some epitope variants, which may impair immunity to strain variants (34).

These findings raised several questions including (i) the overall CD4+ and CD8+ T-cell epitope variation rate in a broader spectrum of viral isolates, (ii) whether conserved epitopes exist that represent generic targets for immunotherapeutic intervention against different viral strains across the world, and (iii) whether random genetic drift or immune selection is driving strain diversification.

Here, we compared more than 300 T-cell epitopes in 168 published EBV strains isolated from a variety of geographical regions and histological sample types. This analysis revealed amino acid exchanges in a large proportion of all CD4+ and CD8+ T-cell epitopes, identified clinically relevant epitopes with no/low inter-strain variability, and provided evidence for immune pressure shaping the evolution of the virus. These results validate our immunoinformatics approach that can readily be adapted to other complex viruses such as CMV and SARS-CoV-2.

## Material and Methods

### Epitope Analysis

All viral strain sequences were downloaded from NCBI. Epitope sequences including their flanking regions were aligned using in-house routines built with MATLAB 9.4 (2018a) (The MathWorks Inc., Natick, MA, USA). If epitopes differed from the B95.8 reference strain, including one or multiple amino acid exchanges, deletions, insertions or strong dissimilarities, they were classified as containing “polymorphisms” either within the epitope, the flanking region, or in both. Results were obtained in absolute or relative numbers, the latter being calculated by dividing individual strain numbers sharing the same epitope variant by the number of total strains that provided sequence data for the respective epitope. Data was further analyzed in GraphPad Prism 7 and MATLAB. Statistical analysis (alpha=0.05) was performed with MATLAB using Chi^2^ goodness of fit test for uniform distribution as well as two-sided binomial test with Bonferroni correction. Allele frequencies were obtained from http://www.allelefrequencies.net.

### Consensus Library

The viral strains sequences alignments using ClustalW algorithm and the resulting consensus library were generated with the MacVector software.

### Assessing Polymorphism Probabilities in Epitope vs. Non-Epitope Regions

Calculation of polymorphism probabilities and adjusting weights were all performed with MATLAB 9.4 (R2018a) as follows:

Definitions:

P_i_: i-th EBV protein

$S_{i}^{p}$ : protein size, as total number of protein P_i_

$S_{i}^{e}$ : total number of amino acids of the epitope regions in protein P_i_

$S_{i}^{\text{ex}}=S_{i}^{p}-S_{i}^{e}$

$\mu_{i}^{e}$ : number of polymorphisms within the epitope regions $\left({\text{S}}_{i}^{e}\right)$ of protein P_i_

$\mu_{\text{i}}^{\text{ex}}$ : number of polymorphisms within the regions excluding epitopes $\left(S_{i}^{\text{ex}}\right)$ of protein P_i_

$P_{i}^{e}$ : probability of polymorphisms occurring within epitope regions of protein P_i_

$P_{i}^{\text{ex}}$ : probability of polymorphisms occurring within the regions excluding epitopes $\left(S_{i}^{\text{ex}}\right)$ of protein P_i_
P^e^: overall probability of polymorphisms occurring within epitope regions of all proteins
P^ex^: overall probability of polymorphisms occurring within the regions excluding epitopes $\left(S_{i}^{\text{ex}}\right)$ of all proteins

$\text{W}{\text{P}}_{i}^{e}$ : weighted probability of polymorphisms occurring within epitope regions of protein P_i_

$\text{W}{\text{P}}_{i}^{\text{ex}}$ : weighted probability of polymorphisms occurring within the regions excluding epitopes $\left(S_{i}^{\text{ex}}\right)$ of protein P_i_
WP^e^: overall weighted probability of polymorphisms occurring within epitope regions of all proteins
WP^ex^: overall weighted probability of polymorphisms occurring within the regions excluding epitopes $\left(S_{i}^{\text{ex}}\right)$ of all proteins

### Calculating Probabilities of Polymorphisms Within Different Protein Regions

The probability of polymorphisms occurring in the epitope regions of one protein P_i_ is the number of polymorphisms within the epitope region divided by the total number of amino acids of the epitope regions. The overall probability of polymorphisms occurring within the epitope regions is the sum of all polymorphisms found in all epitope regions divided by the total number of amino acids of all the epitope regions. The same argument applies to the non-epitope regions.


$${\text{P}}_{i}^{e}=\frac{\mu_{i}^{e}}{s_{i}^{e}};\;P^{e}=\frac{\Sigma_{i}\;\mu_{i}^{e}}{\Sigma_{i}\;s_{i}^{e}}$$

$$P_{i}^{\text{ex}}=\frac{\mu_{i}^{ex}}{s_{i}^{ex}};\;{\text{P}}^{\text{ex}}=\frac{\Sigma_{i}\;\mu_{i}^{ex}}{\Sigma_{i}\;s_{i}^{ex}}$$

### Adjusting the Weights of the Proteins´ Individual Probabilities for the Overall Analysis According to Epitope and Protein Size

The weighted probability of polymorphisms occurring within epitope regions of protein P_i_ is the probability of polymorphisms occurring within epitope regions of protein Pi weighted with the ratio of the number of amino acids of the epitope region in protein P_i_ and the total number of amino acids of the epitope regions of all proteins. This coincides with number of polymorphisms within the epitope regions of protein Pi divided by the total number of amino acids of the epitope regions of all proteins. The same argument applies to the non-epitope regions.


$$\text{W}{\text{P}}_{i}^{e}={\text{P}}_{i}^{e}\;\frac{s_{i}^{e}}{\Sigma_{n}\;s_{n}^{e}}=\frac{\mu_{i}^{e}}{s_{i}^{e}}\frac{s_{i}^{e}}{\Sigma_{n}\;s_{n}^{e}}=\frac{\mu_{i}^{e}}{\Sigma_{n}\;s_{n}^{e}}$$

$$\text{W}{\text{P}}_{i}^{ex}={\text{P}}_{i}^{ex}\;\frac{s_{i}^{ex}}{\Sigma_{n}\;s_{n}^{ex}}=\frac{\mu_{i}^{ex}}{s_{i}^{ex}}\frac{s_{i}^{ex}}{\Sigma_{n}\;s_{n}^{ex}}=\frac{\mu_{i}^{ex}}{\Sigma_{n}\;s_{n}^{ex}}$$

With these definitions, the overall probability of polymorphisms occurring within epitope regions of all proteins coincides with the sum of the weighted probabilities with respect to all proteins. The same argument applies to the non-epitope regions. This means that the weighted probabilities decompose the total probability of polymorphisms within an epitope or non-epitope region according to the protein sizes.


$$\Sigma_{i}\;WP_{i}^{e}=\frac{\Sigma_{i}\;\mu_{i}^{e}}{\Sigma_{i}\;s_{i}^{e}}=P^{e};\;\Sigma_{i}\;WP_{i}^{ex}=\frac{\Sigma_{i}\;\mu_{i}^{ex}}{\Sigma_{i}\;s_{i}^{ex}}=P^{ex}$$

### Kolmogorov-Smirnov Test

The Kolmogorov-Smirnov Test was performed including each data point of the different EBV strains for all 32 EBV proteins using weight-adjusted probabilities.

For each protein variant, $\text{W}{\text{P}}_{i}^{e}\text{ and }WP_{i}^{ex}$ were calculated and the two sets of data were analyzed for statistical significance using the Kolmogorov-Smirnov test using MATLAB 9.4 (R2018a).

## Results

### Geographical and Tissue Origin of EBV Strains

In this analysis of T-cell epitope polymorphisms, genomic sequences of 168 distinct EBV strains from various geographical regions and tissues were included (**Table S1** and **Figure S1A**). Most of the strains had been isolated from donors in Asia (n=58) and Africa (n=42), fewer had been obtained from individuals in North America (n = 28), Australia (n=21), Europe (n=9), and South America (n=9). In accordance with their worldwide prevalence (35), the vast majority of all strains from the different geographical regions were classified as type 1, except for African virus isolates of which 25% were type 2. Furthermore, genomic sequences from almost one third of all Asian strains were recombined or incomplete and classified neither as type 1, nor as type 2. One type 2 strain sample was of unknown origin (**Figure S1A**).

Most strains had been isolated from malignant tissues of epithelial (e.g. nasopharyngeal, gastric and lung carcinomas) or lymphoid origin (e.g. Burkitt lymphoma, PTLD, Hodgkin lymphoma). About one third of the viral strains had been isolated from non-malignant tissues from donors from different parts of the world, including PBMC of patients with infectious mononucleosis (IM) and healthy virus carriers, as well as spontaneous LCL (sLCL) and saliva (**Figure S1B**).

### Frequency of Polymorphisms in CD4+ and CD8+ T-Cell Epitopes

This collection of viral strains from diseased and healthy tissues from around the world was used to assess polymorphisms in EBV-specific T-cell epitopes. To this end, we compared amino acid sequences of 185 CD8+ T-cell epitopes and 120 CD4+ T-cell epitopes that had been retrieved from literature and also included some unpublished CD4+ T-cell epitopes identified by our group (34, 36). Since amino acid sequences flanking an epitope can impact its antigenicity, flanking regions (FR) were defined as five N- and five C-terminal amino acids immediately adjacent to the epitope and were also included in this analysis. Most of the CD4+ (72%) and almost half (46%) of the CD8+ T-cell epitopes were derived from latent cycle antigens. The distribution of T-cell epitopes across EBV antigens is displayed in **Table S2**.

A list of all CD4+ and CD8+ T-cell epitopes and their flanking regions as well as their variants in the analyzed EBV strains is shown in **Table S3**. Because some viral genome sequences are still incomplete, not all epitopes could be analyzed in all viral strains, especially when located close to or within DNA repeats. However, most epitopes were analyzed in more than 150 viral strains.

**Figure 1:**
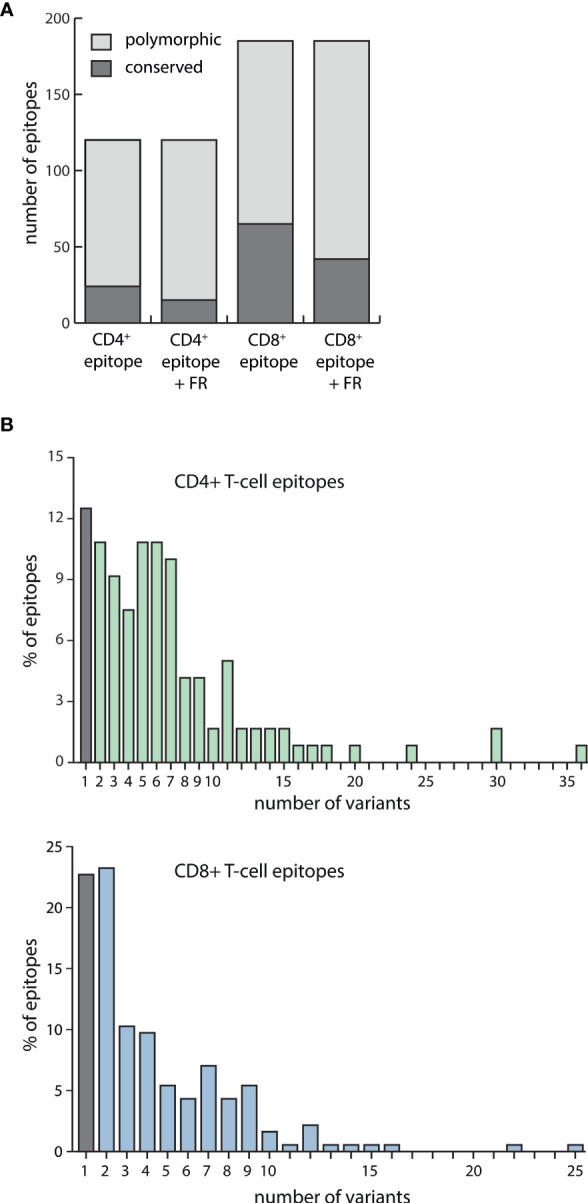
EBV-specific CD4+ and CD8+ T-cell epitope variability among strain variants. **(A)** Amino acid sequences of 120 EBV-specific CD4+ T-cell epitopes and 185 CD8+ T-cell epitopes, either alone or including their flanking regions (FR), were assessed for polymorphisms in 168 EBV strains. Exchanges, insertions, and deletions of single or several amino acids were regarded as polymorphisms. Depicted is the number of epitopes +/- their flanking regions that were found to be conserved or polymorphic among the analyzed viral strains. **(B)** For each of the 120 CD4+ (upper graph) and 185 CD8+ (lower graph) T-cell epitopes including their flanking regions, the number of sequence variants among 168 EBV strains was determined.

Of the 185 CD8+ T-cell epitopes, 65 were conserved in all viral strains. When taking the flanking regions also into account, this number dropped to 42. Accordingly, less than 25% of all CD8+ epitopes plus flanking regions were found to be conserved in all viral strains examined. Of the 120 CD4+ T-cell epitopes, 24 were found to be conserved and this number dropped to 15, or 12.5%, when flanking regions were included (**Figure 1A**).

Polymorphic CD4+ T-cell epitopes were found to have a mean number of 7 and a maximum number of 36 variants, while CD8+ epitopes varied less with a mean number of 4 and a maximum number of 25 variants (**Figure 1B**). Detailed polymorphism analysis for every CD4+ and CD8+ T-cell epitope is provided in **Table S3**.

### Epitope Variability Within EBV Strains

**Figure 2:**
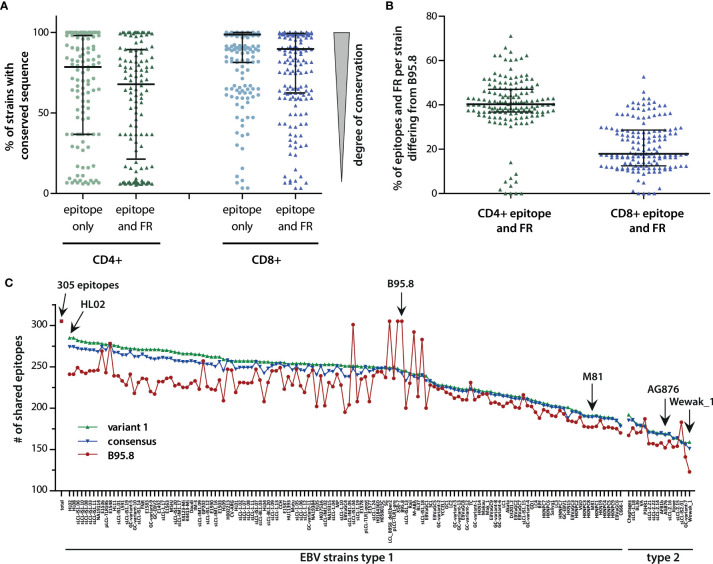
Variability of T-cell epitopes among viral strains. **(A)** Percentage of EBV strains with conserved sequence for each CD4+ or CD8+ T-cell epitope, with and without flanking regions (FR). Each dot represents one epitope. Median including 25% and 75% percentiles are shown. **(B)** Percentage of epitopes including flanking regions, in which a given strain differs from B95.8. Median (black bar) with interquartile ranges (grey bars) are shown. **(C)** Number of epitopes shared by viral strains with epitope libraries. Shown in red are the number of epitopes plus flanking regions shared by individual viral strains with B95.8. Depicted in green and blue are the number of epitopes including flanking regions shared by individual strains with the “variant 1” and “consensus” libraries, respectively. “Variant 1” consists of those epitope variants most frequently found in all viral strains analyzed (epitope variant1, **Table S3**). The “consensus” library of T-cell epitopes was established by selecting those amino acids for each amino acid position of the epitopes that were found most frequently in all viral isolates (**Table S5**). For incompletely sequenced strains, the number of epitopes was normalized (number of shared epitopes multiplied by 305 and divided by the number of epitopes analyzed in this strain). Besides B95.8, the NPC-derived type 1 strain M81, the prototypic type 2 strain AG876, and the in all analyses most incongruent strain Wewak_1 are marked with arrows.

These results suggested that only a limited number of conserved T-cell epitopes represent generic immune targets against all viral variants. However, several epitopes were conserved or differed in only a small number of viral isolates and thus might still be effective against a broad set of viral strains. Therefore, median sequence variations in CD4+ and CD8+ T-cell epitopes were assessed by calculating the percentage of strains in which each epitope was found to be conserved with respect to the B95.8 sequence (**Figure 2A**). On average, any given CD4+ T-cell epitope was estimated to be conserved in almost 80% of viral strains. If flanking regions were considered, CD4+ T-cell epitopes were conserved in almost 70% of all viral strains. In the case of CD8+ T-cell epitopes, the degree of conservation was even higher. On average, any given CD8+ T-cell epitope was found to be conserved in almost 99% of all viral strains, and this number dropped to 90% when flanking regions were included into the analysis. While these numbers implied that T-cell responses against these epitopes may be effective against infection with almost all viral strains, the high level of scattering suggested that strains with vastly disparate sets of T-cell epitopes may exist. To identify such variants that are possibly refractory to B95.8 antigen sequence-based immunotherapy, viral strains were ranked according to the number of epitope variants they contained. This analysis revealed that in any given strain, on average 40% of all CD4+ and 20% of all CD8+ T-cell epitopes including their flanking regions differed from the B95.8 sequence (**Figure 2B**). Strains that differed greatly from B95.8 consensus were mostly of type 2. With more than 70% divergence in CD4+ and more than 50% in CD8+ T-cell epitopes, the most divers strain was found to be the type 2 strain Wewak. As there were only few viral isolates that shared all or almost all epitopes with B95.8, this analysis indicated that B95.8 may not represent the ideal reference strain for T-cell epitopes. To investigate this in more detail, the number of epitopes plus flanking regions shared by viral strains with B95.8 was determined (**Figure 2C**). Five strains shared almost all 305 epitopes with B95.8 but this number declined to 163 within the group of EBV type 1, and ranged between 191 and 126 in type 2 strains with Wewak_1 showing the least overlap. To explore the possibility of higher and more balanced coverage, two epitope libraries were designed. The “variant 1” library consisted of those epitope and flanking region variants that were most frequently found in all strains analyzed (epitope variant 1; **Table S3**). The “consensus” library was generated by selecting those amino acids for each position of a given epitope plus flanking regions that were most frequently found at this position in viral isolates (**Table S5**). Next, the number of T-cell epitopes shared by a given viral strain with these designer libraries was determined (**Figure 2C**). Compared to B95.8, an overall higher degree of congruity and a narrower range was noted for both libraries (average number of epitopes shared by viral strains with B95.8: 217, with consensus library: 230, with variant 1 library: 235; range B95.8: 123-305, consensus library: 151-278, variant 1 library: 158-285). Due to the high proportion of type 1 strains in this collection, congruity was highest for type 1 strains, whereas NPC-derived type 1 and type 2 strains clustered at the lower end. Thus, the separation of strains observed in principal component analyses (5) was also reflected at the T-cell epitope level (**Figure 2C**).

### Immune-Driven Epitope Diversification

**Figure 3:**
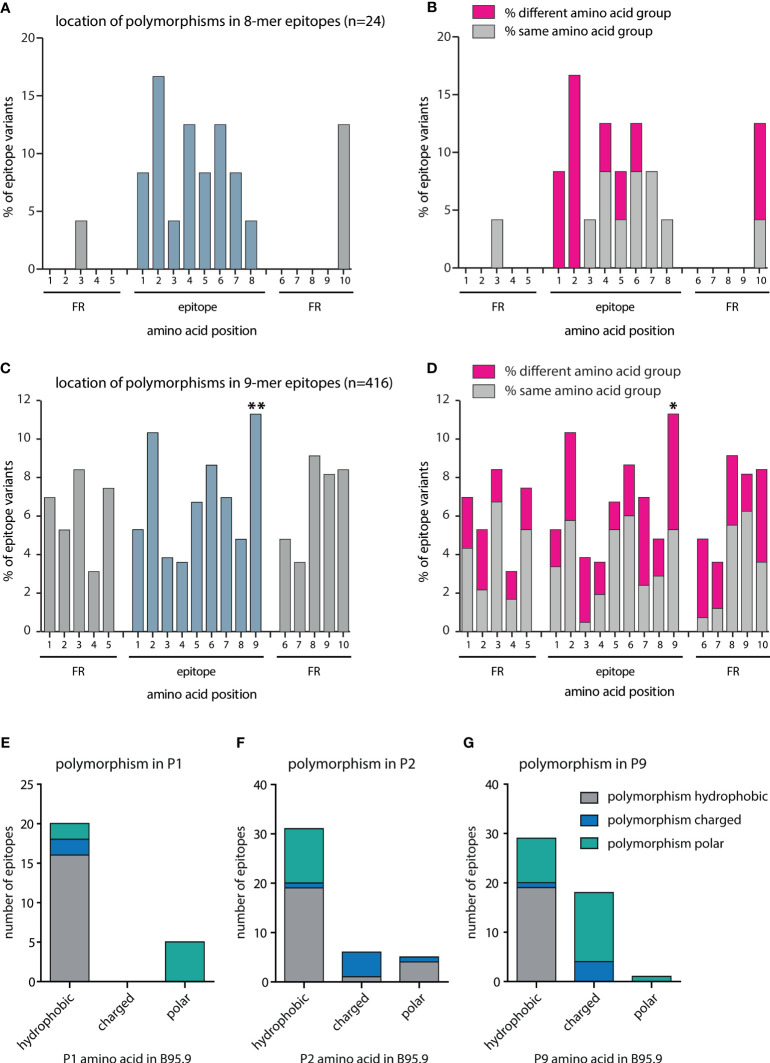
Quantitative and qualitative analysis of amino acid exchanges in CD8+ T-cell epitopes and epitope flanking regions. All polymorphisms in octamer and nonamer CD8+ T-cell epitopes and their flanking regions were included in this analysis. The number of polymorphisms was determined separately for each amino acid position in all variants. Depicted are the frequencies of polymorphisms for each amino acid position in **(A)** octamers plus flanking regions (n=24), and **(C)** nonamers plus flanking regions (n=416). Chi^2^-test goodness of fit test for uniform distribution was performed using MATLAB: **(A)** Chi^2^-value 25,45 (*p* = 0.0851), **(C)** Chi^2^-value 71,15 (*p* < 0.00001). For each position, a two-sided binomial test was performed using Bonferroni adjustment. P-values were indicated as follows *p ≤ 0.002; **p ≤ 0.0002. **(B, D)** Amino acids were divided into the subgroups charged (D, E, K, R), polar (C, H, N, Q, S, T, W, Y), and hydrophobic (A, F, G, I, L, M, P, V). For each position, the percentage of polymorphisms was determined that resulted in a change of the amino acid subgroup. In nonamer epitopes, the number of hydrophobic, charged, and polar amino acids present at positions one **(E)**, two **(F)**, and nine **(G)** was determined and the number of amino acid exchanges resulting in the same or a different subgroup defined.

To investigate whether these sequence variations were caused by random genetic drift or by an immune-driven selection process, the location of amino acid exchanges in octamer and nonamer CD8+ T-cell epitopes and their flanking regions was investigated. In the case of the 7 octamer epitopes and their variants (n=24), most polymorphisms were located within the epitopes and barely within the flanking regions. Furthermore, most amino acid exchanges were noted at position 2 of the epitope, which is required for anchoring the peptide to the MHC binding pocket (**Figure 3A**). To assess whether these polymorphisms changed the chemical characteristics of the peptides and thereby impacted on peptide/MHC interactions, amino acids were divided into charged, polar, and hydrophobic subgroups. All polymorphisms at position 1 and 2 of the epitope resulted in a change of the subgroup (**Figure 3B**). While these results were indicative of an immune-driven diversification process, the low number of 8-mer epitopes (n=24) precluded statistical validation.

Therefore, the same analysis was performed with the 78 polymorphic nonamer epitopes and their variants (n=416) listed in **Table S3**. In this case, a statistically significant increase in amino acid exchanges at position nine was noted (**Figures 3C, D**). Most peptides that bind to MHC I have a hydrophobic (or sometimes basic) anchor residue at the carboxy terminus. Although not reaching statistical significance, frequent amino acid exchanges were also noted at position two, which is another important anchor residue for several HLA class I alleles. While amino acid substitutions at position 1 (**Figure 3E**), which is not critical for binding to most HLA class I molecules, were mostly of the same subgroup, amino acids at positions 2 (**Figure 3F**) and 9 (**Figure 3G**) were often replaced by members of different subgroups. Overall, approximately 50% of the amino acids at P9 were exchanged for amino acids of other subgroups. Approximately one third of the hydrophobic amino acids present in the B95.8 epitopes were exchanged for polar or charged amino acids and the majority of charged amino acids at P9 were exchanged for amino acids of the polar subgroup. Such amino acids are not favored by most MHC class I alleles at this position. Almost half of the polymorphisms at position two resulted in a change in the amino acid subgroup. In addition, amino acids at positions 3 and 7, considered secondary anchor residues for several HLA alleles, were exchanged for members of other subgroups in the majority of cases. Likewise, polymorphisms in the first amino acid downstream of the epitope (position 6 FR), which impacts on proteasomal cleavage, often altered the amino acid subgroup (**Figure 3D**). Collectively, these findings indicated that the polymorphisms in epitopes are likely to have consequential effects on the immune recognition.

### Polymorphisms Are Enriched in Epitope *Versus* Non-Epitope Sequences

**Figure 4:**
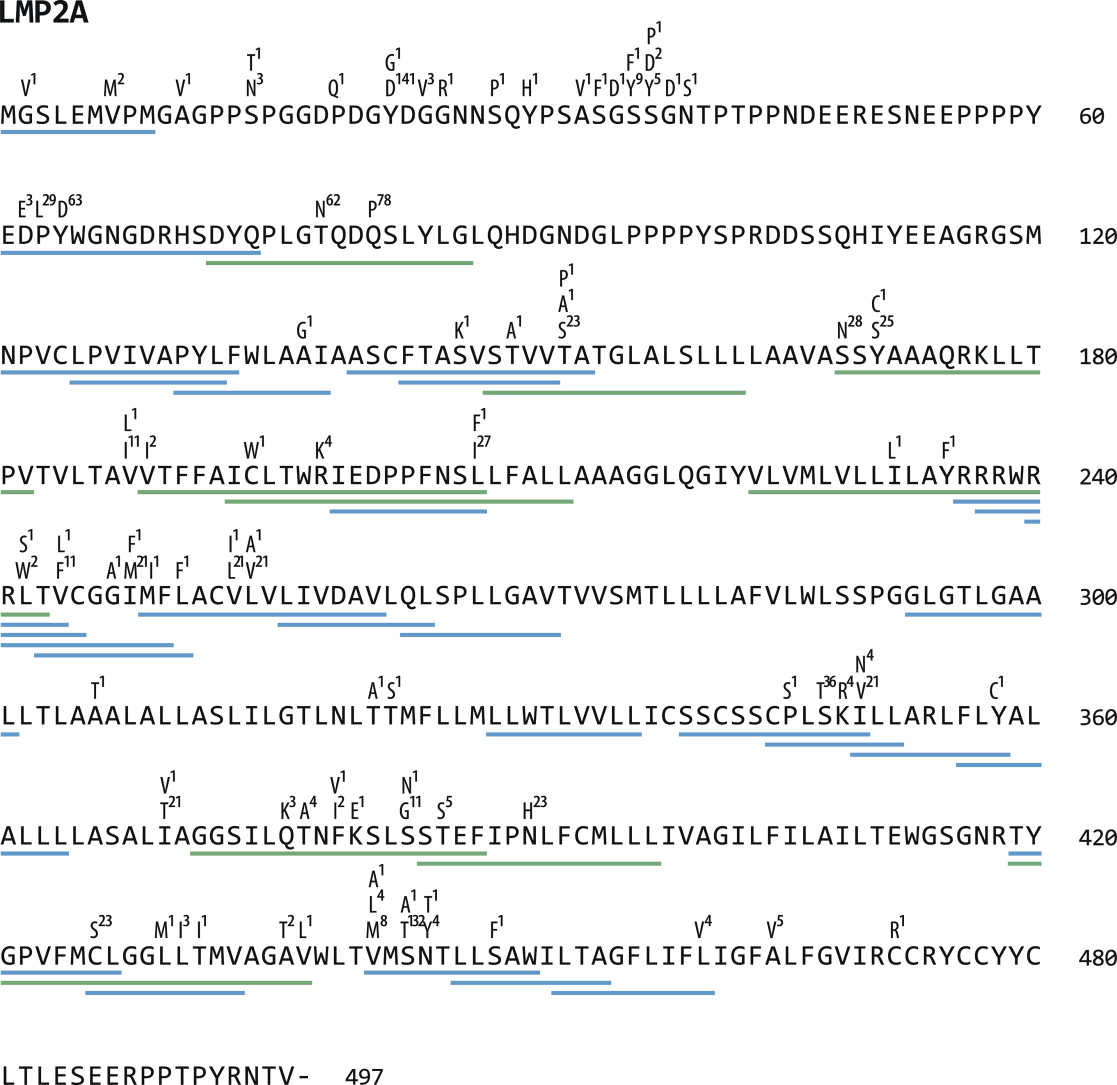
Location of polymorphisms and T-cell epitopes in LMP2A. LMP2A protein sequences of 155 viral strains were aligned and polymorphisms identified using Clustal Omega software (https://www.ebi.ac.uk/Tools/msa/clustalo/). All identified amino acid exchanges and the number of strains in which they occurred are displayed above the LMP2A reference sequence from B95.8. Underneath the sequence, green (CD4+) and blue (CD8+) lines indicate the location of T-cell epitopes (further details can be found in **Table S3**).

**Figure 5:**
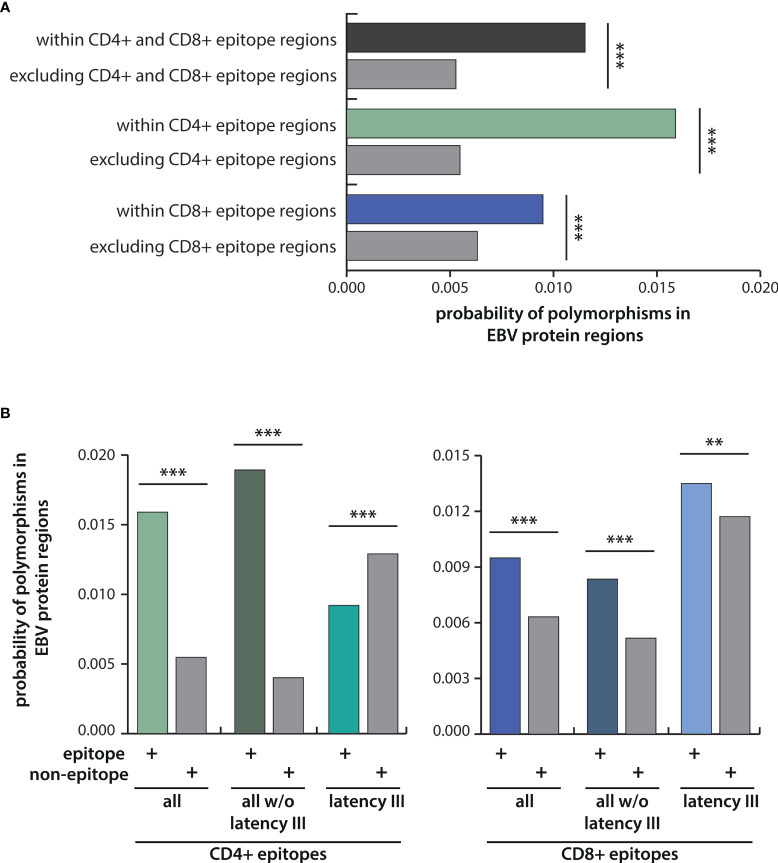
Probabilities of polymorphisms occurring within epitope regions in comparison to the rest of the protein. **(A)** Depicted are the probabilities of amino acid exchanges occurring within T-cell epitopes in comparison to the rest of the protein for all CD4+ and CD8+ T-cell epitopes taken together, and for CD4+ and CD8+ T-cell epitopes separately. **(B)** Probabilities for polymorphisms in CD4+ (left) and CD8+ (right) T-cell epitopes were further analyzed for latency III antigens (EBNA2, EBNA-LP, EBNA3A, B, C) and for all antigens except latency III antigens. Statistical significance was tested with the Kolmogorov-Smirnov test, which was performed including each data point of the different EBV strains for all 32 EBV proteins using weight-adjusted probabilities (for details see Methods section). **p < 0.05; ***p < 0.005.

To investigate the role of immune pressure as a driving force behind viral strain diversification, we assessed the locations of T-cell epitopes and polymorphisms in proteins. As exemplified for LMP2A in **Figure 4**, polymorphic regions and epitopes often colocalized, especially when polymorphisms occurred in a large number of strains. A systematic analysis of polymorphism rates in epitope and non-epitope sequences in all 32 viral proteins, in which T-cell epitopes had been identified, demonstrated that polymorphisms were significantly enriched in both CD4+ and CD8+ T-cell epitopes (p-value <0.005) (**Figure 5A**). The probability that any given amino acid was affected by a polymorphism and belonged to a CD4+ T-cell epitope was found to be almost threefold higher than in regions of the protein that are not known to be targeted by T cells. For CD8+ T-cell epitopes, this ratio was found to be 1,5-fold increased. When the epitopes were subdivided in latency type III antigens (EBNA2, EBNA-LP, and EBNA3 family) and all antigens except latency type III antigens, polymorphisms in CD4+ T-cell epitopes in the latter group were further enriched, but reduced in epitopes in latency type III antigens (**Figure 5B**). Because similar numbers of lytic cycle (n=34) and latency type III-associated (n=35) CD4+ T-cell epitopes have been analyzed (**Table S2**), this discrepancy is unlikely to be caused by differences in sample sizes. Furthermore, a similar trend, albeit less pronounced, became apparent for CD8+ T-cell epitopes. These results identify immune pressure as driver in EBV strain diversification, except for CD4+ T-cell epitopes in latency type III antigens, which appear to be under negative selection.

## Discussion

To study the clinical implications of EBV strain heterogeneity, we compared EBV T-cell epitopes in more than 150 viral strains derived from normal and diseased tissues and from different geographical regions. Approximately 30% of all CD8+ and 80% of all CD4+ T-cell epitopes were found to be polymorphic and these numbers further increased when the epitope flanking regions were included. However, one has to keep in mind that many of the published CD4+ T-cell epitopes were defined using overlapping peptide libraries of 15 or 20 amino acids in length (**Table S3**). The core sequence of CD4+ T-cell epitopes, however, usually encompasses only 9 amino acids. Consequently, some of the identified polymorphisms may reside outside the core epitope or its flanking regions and have led to an over-estimation in the number of polymorphisms in CD4+ T-cell epitopes.

In addition, several of the analyzed viral strains were derived from tumor cell lines in which mutations in T-cell epitopes may have accumulated due to genetic instability and/or immune pressure and thereby contributed to these high polymorphism rates. This, however, appears unlikely because rare (tumor-specific) epitopes were not found to be enriched in strains isolated from tumor cells (**Table S3**).

On average, viral strains differed from B95.8 in 40% of all CD4+ and 20% of all CD8+ T-cell epitopes including flanking regions and variability extended over a broad range, challenging the role of B95.8 as reference strain for T-cell epitopes. Polymorphisms in T-cell epitopes have previously been implicated as potential reason for low clinical efficacy of B95.8 LCL-stimulated T-cell preparations in some PTLD patients and their failure to recognize spontaneous LCL from these patients (37–45). Although LCL usually present multiple viral antigens on MHC class I and II, including some that were probably shared between B95.8 and the endogenous virus of the patients, the CTL response is often dominated by few epitopes so that even polyclonal EBV-specific T-cell preparations may be oligoclonal in specificity (46–48). Consequently, epitope libraries containing the most frequent epitope variants or designed by selecting those amino acids most frequently found at a given position of an epitope in all EBV strains, may constitute better targets for diagnostic and therapeutic purposes when the infecting strain is unknown. Alternatively, BAC technology may be used to generate viral strains with higher and more balanced epitope coverage such as HL02 (**Figure 2C**) and used for T-cell stimulation instead of B95.8 (49).

Despite the overall high variability, several T-cell epitopes were found to be conserved in all strains analyzed, including some immunodominant EBV antigens (**Table S4**), such as the GLC-epitope derived from BMLF1, or the YVL-epitope derived from BRLF1, that may represent generic targets for clinical application. The reason for this conservation is not known, but as described for other viruses, these epitopes may be located in regions critical for protein function in which escape comes at the cost of viral fitness (50). By further analogy to other pathogens, the development of such escape mutants would also be unlikely to occur if T-cell responses against these epitopes had beneficial effects on virus pathobiology or negligible effects on viral transmission (51, 52).

Previous analyses demonstrated that amino acid exchanges in epitopes and their flanking regions can retain, reduce, or abolish T-cell recognition [(53) and references therein]. Polymorphisms within or in close proximity to epitopes may affect T-cell recognition in various ways, e.g. by altering antigen processing, presentation, and binding to MHC and the T-cell receptor (54–58). Therefore, the impact of single amino acid exchanges on the T-cell response is generally difficult to predict. In the case of CD8+ T-cell epitopes, stable binding to MHC class I molecules depends on distinct anchor residues, usually the amino acids at position 2 and at the C-terminus (59). These residues were often found to be polymorphic and, in many cases, exchanged for amino acids from different biochemical subgroups. Similarly, polymorphisms in the first residue in the C-terminal flanking region often resulted in a switch in the amino acid subgroup. The C-terminus of most MHC I ligands is generated by proteasomal cleavage and although amino acid preferences downstream of the scissile peptide bond seem to vary between model proteins, polar amino acids, particularly serine, were recently found to be enriched at this position (60, 61). Although still requiring experimental verification, these finding are suggestive of immune escape due to impaired epitope generation and MHC binding. However, due to differences in HLA genotype prevalence around the world, we cannot exclude that some of the variants may represent immunogenic epitopes in association with certain HLA molecules. Furthermore, no such analysis was performed for CD4+ T-cell epitopes because only few core sequences had been mapped. In addition, CD4+ T-cell epitopes usually contain four anchor residues and the contribution of single anchor amino acids to MHC class II binding is still poorly defined. Nevertheless, the notion of antigenic drift was further substantiated when polymorphism rates in epitopes and non-epitope sequences were compared. Amino acid exchanges were significantly more frequent in CD4+ and CD8+ T-cell epitope sequences as compared to the rest of the proteins. The probability of a given polymorphism to be part a CD4+ T-cell epitope versus non-epitope region was almost threefold higher. For CD8+ T-cell epitopes, this probability was 1.5-fold increased. These results infer an important role of T-cell responses in driving virus diversification.

The question whether adaptive immunity is shaping the evolution of the virus is longstanding and has engendered controversial debates in the past following the discovery that EBV type 1 strains from highly HLA-A*11 positive Asian populations consistently display variations within two HLA-A*11-restricted, immunodominant CD8+ T-cell epitopes derived from EBNA3B, which render these epitope variants non-immunogenic *in vivo* (29, 62, 63). Such epitope loss would confer a selective advantage to strain variants in this particular host community and was interpreted by some as evidence of immune selection. Others, however, inferred random genetic drift because polymorphisms in these as well as additional epitopes from the EBNA3 family of proteins coherently mapped onto the phylogenetic tree of Chinese EBV type 1 strains and thereby implied a common viral origin of these strains. Moreover, such epitope-loss mutations were not replicated at other epitope loci (11, 33, 64, 65), which is consistent with recent findings showing that most positively selected codons in EBNA3 genes map to regions outside established CD8+ T-cell epitopes (5).

In contrast to these studies, which were often performed on a limited number of epitopes and viral isolates, the present analysis of 305 epitopes in more than 150 strains provides clear evidence that the T-cell response is driving virus diversification. However, immune pressure seems to shape the virus in opposite ways. While polymorphisms were generally found to be enriched in CD4+ and CD8+ T-cell epitopes, CD4+ T-cell epitopes in latency type III antigens appear to be under negative selection pressure, resulting in sequence diversification and conservation, respectively. While diversification is pointing towards immune evasion of the virus, the conservation of epitopes within the most variable viral proteins that are considered immunodominant targets of the virus-specific T-cell responses, appears more difficult to reconcile. Besides functional constraints, low variability in latency type III epitopes may have evolved in benefit of the virus. Since expression of these antigens is normally restricted to newly infected B cells, their conservation may ensure that any aberrantly expanding cell population expressing these antigens will be efficiently eliminated by specific T cells and not kill the host, as proposed earlier (66). In this case, however, a stronger negative selection pressure on epitopes recognized by cytotoxic CD8+ T cells would have been expected. In an alternative scenario, immune responses against these antigens may facilitate access of the virus to the memory compartment, the actual site of viral persistence. According to the germinal center model, EBV infects naïve B cells *in vivo* and expression of the growth program enables EBV-infected blasts to migrate into follicles of secondary lymphoid organs where they switch to latency type I and leave the follicle as resting memory B cells (66). This transition in latency type is thought to depend on exogenous signals provided by activated CD4+ T cells (35, 66, 67), such as IL21 and soluble CD40L (67–69). The conservation of CD4+ T-cell epitopes in type III latency antigens may increase the likelihood of B cells infected with different viral strains for encountering and receiving T-cell help and thereby foster coinfections. Of note, increasing evidence suggests that coinfections are the rule, rather than the exception (70, 71).

If correct, this coinfection model would posit that persistent infection is a much more dynamic process than hitherto thought. For verifying this model, several predictions can be made and tested experimentally. For instance, do polymorphisms in latency type III epitopes have a milder immunological phenotype, i.e. lower impact on T-cell recognition, than epitopes under positive selection, do co-resident viral strains express mostly shared sets of latency type III epitopes, and do CD4+ T cells specific for these antigens form an integral part of the follicular CD4+ T-cell compartment? Ultimately, these studies may contribute to a better understanding of the biology of this virus and its intricate relationship with the human host, and inform on the targets best suited for clinical intervention.

## Data Availability Statement

The original contributions presented in the study are included in the article/**Supplementary Material**. Further inquiries can be directed to the corresponding author.

## Author Contributions

UB and JM conceived and designed the study. AC and RP collected and analyzed sequences. AC and MD performed the statistical analyses. KW provided information on HLA genotypes. H-JD contributed to the acquisition, analysis, and interpretation of data. AC and JM wrote the paper and all authors made substantial contributions to data analysis and interpretation, manuscript editing, review and approval.

## Funding

Funding was received by DZIF, the German Center for Infection Research (TTU 07.0804).

## Conflict of Interest

The authors declare that the research was conducted in the absence of any commercial or financial relationships that could be construed as a potential conflict of interest.

## Publisher’s Note

All claims expressed in this article are solely those of the authors and do not necessarily represent those of their affiliated organizations, or those of the publisher, the editors and the reviewers. Any product that may be evaluated in this article, or claim that may be made by its manufacturer, is not guaranteed or endorsed by the publisher.
