# Supplementary material for: Immunoinformatic Analysis Reveals Antigenic Heterogeneity of Epstein-Barr Virus Is Immune-Driven
Source: Front Immunol. 2021 Dec 16;12:796379. doi: 10.3389/fimmu.2021.796379 (PMC8716887; doi:10.3389/fimmu.2021.796379)
Supplement: Supplementary file 1 [file DataSheet_1.docx]

**Supplemental Figure S1**


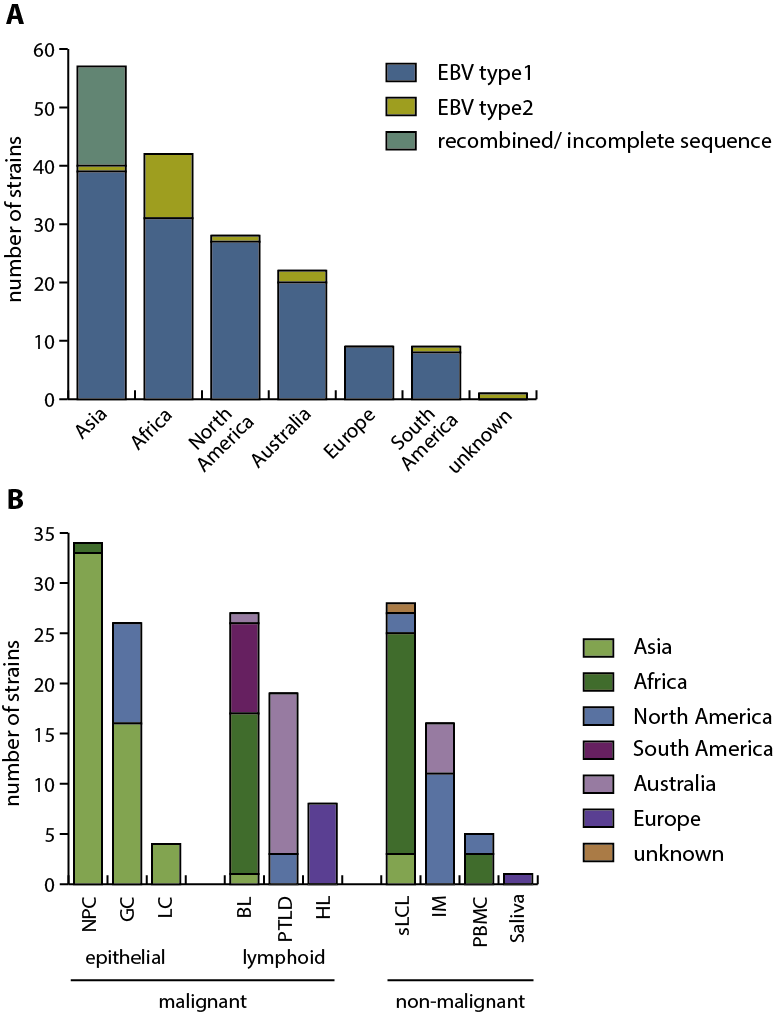


**Fig S1: Origin of viral isolates included in this study.**

(A) Type1/2 classification and geographical origin of EBV strains included in this analysis. Depicted is the number of viral isolates from different geographical regions and their classification as type 1 or type 2. Due to recombination events or incomplete sequence information, some viral isolates could not be subtyped. (B) Geographical provenience of the analyzed EBV strains and tissue samples from which they were isolated. NPC, nasopharyngeal carcinoma; GC, gastric carcinoma; LC, lung cancer; BL, Burkitt lymphoma; PTLD, post-transplant lymphoproliferative disease; HL, Hodgkin lymphoma; IM, infectious mononucleosis; sLCL, spontaneous lymphoblastoid cell line.
