# Supplementary material for: Immunoinformatic Analysis Reveals Antigenic Heterogeneity of Epstein-Barr Virus Is Immune-Driven
Source: Front Immunol. 2021 Dec 16;12:796379. doi: 10.3389/fimmu.2021.796379 (PMC8716887; doi:10.3389/fimmu.2021.796379)
Supplement: Supplementary file 2 [file Table_1.docx]

**Table S1. List of viral strains**

| # | Name | EBV subtype | Tissue | Geographic origin | GenBank accession # | Reference |
| --- | --- | --- | --- | --- | --- | --- |
| 1 | **AG876** | 2 | BL | Africa (Ghana) | DQ279927.1 | (1) |
| 2 | **Akata** | 1 | BL | Asia (Japan) | KC207813.1 | (2) |
| 3 | **BL36** | 2 | BL | North Africa | LN827557.2 | (3) |
| 4 | **BL37** | 1 | BL | Africa | LN827526.1 | (3) |
| 5 | **Cheptages** | 2 | BL | Africa (Kenya) | LN827556.1 | (3) |
| 6 | **CCH** | 1 | BL | South America (Brazil) | KP968257.1 | (4) |
| 7 | **CV-ARG** | 1 | BL | South America (Argentina) | KR063343.1 | (4) |
| 8 | **Daudi** | 1 | BL | Africa (Kenya) | LN827545.1 | (3) |
| 9 | **FNR** | 1 | BL | South America (Brazil) | KR063345.1 | (4) |
| 10 | **H002213** | 1 | BL | Africa (Ghana) | KP968264.1 | (4) |
| 11 | **H03753A** | 1 | BL | Africa (Ghana) | KR063342.1 | (4) |
| 12 | **H018436D** | 1 | BL | Africa (Ghana) | KP968262.1 | (4) |
| 13 | **H058015C** | 1 | BL | Africa (Ghana) | KP968263.1 | (4) |
| 14 | **HU11393** | 1 | BL | Africa (Ghana) | KP968261.1 | (4) |
| 15 | **Jijoye** | 2 | BL | Africa (Nigeria) | LN827800.1 | (3) |
| 16 | **Mak_1** | 1 | BL | Africa (Kenya) | LN824203.1 | (3) |
| 17 | **Makau** | 1 | BL | Africa (Kenya) | LN827551.1 | (3) |
| 18 | **MP** | 1 | BL | South America (Brazil) | KP968258.1 | (4) |
| 19 | **Mutu** | 1 | BL | Africa (Kenya) | KC207814.1 | (2) |
| 20 | **P3HR1** | 2 | BL | Africa (Nigeria) | LN827548.2 | unpublished |
| 21 | **Raji** | 1 | BL | Africa (Nigeria) | KF717093.1 | unpublished |
| 22 | **RPF** | 1 | BL | South America (Brazil) | KR063344.1 | (4) |
| 23 | **SCL** | 1 | BL | South America (Brazil) | KP968259.1 | (4) |
| 24 | **SG** | 1 | BL | South America (Argentina) | KT001103.1 | (4) |
| 25 | **VA** | 2 | BL | South America (Argentina) | KT001102.1 | (4) |
| 26 | **VGO** | 1 | BL | South America (Brazil) | KP968260.1 | (4) |
| 27 | **Wewak_1=Wewak_2** | 2 | BL | Australia (Papua New Guinea) | LN827544.1 | (3) |
| 28 | **HL01** | 1 | HL | Europe (UK) | LN824226.1 | (3) |
| 29 | **HL02** | 1 | HL | Europe (UK) | LN827546.1 | (3) |
| 30 | **HL04** | 1 | HL | Europe (UK) | LN827564.1 | (3) |
| 31 | **HL05** | 1 | HL | Europe (UK) | LN824204.1 | (3) |
| 32 | **HL08** | 1 | HL | Europe (UK) | LN824225.1 | (3) |
| 33 | **HL09** | 1 | HL | Europe (UK) | LN827522.1 | (3) |
| 34 | **HL11** | 1 | HL | Europe (UK) | LN827524.1 | (3) |
| 35 | **L591** | 1 | HL-cell line | Europe (Germany) | LN827523.1 | (3) |
| 36 | **EBVaGC1** | 1 | GC | Asia (China, Beijing) | KT273942.1 | (5) |
| 37 | **EBVaGC2** | 1 | GC | Asia (China, Beijing) | KT273943.1 | (5) |
| 38 | **EBVaGC3** | 1 | GC | Asia (China, Beijing) | KT254013.1 | (5) |
| 39 | **EBVaGC4** | 1 | GC | Asia (China, Beijing) | KT273944.1 | (5) |
| 40 | **EBVaGC5** | 1 | GC | Asia (China, Beijing) | KT273945.1 | (5) |
| 41 | **EBVaGC6** | 1 | GC | Asia (China, Beijing) | KT273946.1 | (5) |
| 42 | **EBVaGC7** | 1 | GC | Asia (China, Beijing) | KT273947.1 | (5) |
| 43 | **EBVaGC8** | 1 | GC | Asia (China, Beijing) | KT273948.1 | (5) |
| 44 | **EBVaGC9** | 1 | GC | Asia (China, Beijing) | KT273949.1 | (5) |
| 45 | **GC-EBV1=GDGC1** | 1 | GC | Asia (China, Guangzhou) | KX674064.1 | (6) |
| 46 | **GC-EBV2=GDGC2** | 1 | GC | Asia (China, Guangzhou) | KX674065.1 | (6) |
| 47 | **GC1=SNU719** | 1 | GC-cell line | Asia (South Korea) | KP735248.1 | (7) |
| 48 | **GC-variant-1** | 1 | GC | Asia (China) | MG021314.1 | (8) |
| 49 | **GC-variant-2** | 1 | GC | Asia (China) | MG021305.1 | (8) |
| 50 | **GC-variant-3** | 1 | GC | Asia (China) | MG021315.1 | (8) |
| 51 | **GC-variant-4** | 1 | GC | Asia (South Korea) | MG021317.1 | (8) |
| 52 | **GC-variant-5** | 1 | GC | Europe (Poland) | MG021308.1 | (8) |
| 53 | **GC-variant-6** | 1 | GC | Asia (South Korea) | MG021307.1 | (8) |
| 54 | **GC-variant-7** | 2 | GC | Asia (South Korea) | MG021312.1 | (8) |
| 55 | **GC-variant-8** | 1 | GC | Asia (Viet Nam) | MG021316.1 | (8) |
| 56 | **GC-variant-9** | 1 | GC | Europe (Poland) | MG021310.1 | (8) |
| 57 | **GC-variant-10** | 1 | GC | Europe (Ukraine) | MG021311.1 | (8) |
| 58 | **GC-variant-11** | 1 | GC | North America (USA) | MG021309.1 | (8) |
| 59 | **GC-variant-12** | 1 | GC | Asia (South Korea) | MG021313.1 | (8) |
| 60 | **GC-variant-13** | 1 | GC | Europe (Poland) | MG021306.1 | (8) |
| 61 | **YCCEL1** | 1 | GC-cell line | Asia (South Korea) | AP015016.1 | (3) |
| 62 | **B95.8** | 1 | IM | North America (USA) | V01555.2 | (9) |
| 63 | **E1492_BCv1** | 1 | IM | North America (USA) | MF547477.1 | (10) |
| 64 | **E1503_BCv1** | 1 | IM | North America (USA) | MF547481.1 | (10) |
| 65 | **E1536_BCv1** | 1 | IM | North America (USA) | MF547461.1 | (10) |
| 66 | **E1548_BCv1** | 1 | IM | North America (USA) | MF547466.1 | (10) |
| 67 | **E1563_BCv1** | 1 | IM | North America (USA) | MF547463.1 | (10) |
| 68 | **E1577_BCv1** | 1 | IM | North America (USA) | MF547489.1 | (10) |
| 69 | **E1578_BCv1** | 1 | IM | North America (USA) | MF547485.1 | (10) |
| 70 | **E1583_BCv1** | 1 | IM | North America (USA) | MF547453.1 | (10) |
| 71 | **E1587_BCv1** | 1 | IM | North America (USA) | MF547457.1 | (10) |
| 72 | **E1590_BCv1** | 1 | IM | North America (USA) | MF547473.1 | (10) |
| 73 | **sLCL-IM1.02** | 1 | sLCL, IM | Australia | LN827596.1 | (3) |
| 74 | **sLCL-IM1.05** | 1 | sLCL, IM | Australia | LN827590.1 | (3) |
| 75 | **sLCL-IM1.09** | 1 | sLCL, IM | Australia | LN827567.1 | (3) |
| 76 | **sLCL-IM1.16** | 1 | sLCL, IM | Australia | LN827799.1 | (3) |
| 77 | **sLCL-IM1.17** | 1 | sLCL, IM | Australia | LN827583.1 | (3) |
| 78 | **LC1** | 1 | LC | Asia (China, Beijing) | KT823506.1 | (11) |
| 79 | **LC2** | 1 | LC | Asia (China, Beijing) | KT823507.1 | (11) |
| 80 | **LC3** | 1 | LC | Asia (China, Beijing) | KT823508.1 | (11) |
| 81 | **LC4** | 1 | LC | Asia (China, Beijing) | KT823509.1 | (11) |
| 82 | **GD1** | 1 | NPC saliva | Asia (China) | AY961628.3 | (12) |
| 83 | **GD2** | 1 | NPC tumor | Asia (China) | HQ020558.1 | (13) |
| 84 | **C666-1** | 1 | NPC cell line | Asia (China, Hong Kong) | KC617875.1 | (14) |
| 85 | **D3201.2** | 1 | NPC | Asia (China) | LN827549.1 | (3) |
| 86 | **HKNPC1** | 1 | NPC tumor | Asia (China, Hong Kong) | JQ009376.2 | (15) |
| 87 | **HKNPC2** | 1 | NPC | Asia (China, Hong Kong) | KF992564.1 | (16) |
| 88 | **HKNPC3** | 1 | NPC | Asia (China, Hong Kong) | KF992565.1 | (16) |
| 89 | **HKNPC4** | 1 | NPC | Asia (China, Hong Kong) | KF992566.1 | (16) |
| 90 | **HKNPC5** | 1 | NPC | Asia (China, Hong Kong) | KF992567.1 | (16) |
| 91 | **HKNPC6** | 1 | NPC | Asia (China, Hong Kong) | KF992568.1 | (16) |
| 92 | **HKNPC7** | 1 | NPC | Asia (China, Hong Kong) | KF992569.1 | (16) |
| 93 | **HKNPC8** | 1 | NPC | Asia (China, Hong Kong) | KF992570.1 | (16) |
| 94 | **HKNPC9** | 1 | NPC | Asia (China, Hong Kong) | KF992571.1 | (16) |
| 95 | **M-ABA** | 1 | LCL, NPC virus | North Africa (Algeria) | LN827527.1 | (3) |
| 96 | **M81** | 1 | NPC | Asia (China, Hong Kong) | KF373730.1 | (17) |
| 97 | **HN1** | recombined | NPC | Asia (China, Hunan) | AB850643.1 | (18) |
| 98 | **HN2** | recombined | NPC | Asia (China, Hunan) | AB850644.1 | (18) |
| 99 | **HN3** | recombined | NPC | Asia (China, Hunan) | AB850648.1 | (18) |
| 100 | **HN4** | recombined | NPC | Asia (China, Hunan) | AB850649.1 | (18) |
| 101 | **HN5** | recombined | NPC | Asia (China, Hunan) | AB850652.1 | (18) |
| 102 | **HN6** | recombined | NPC | Asia (China, Hunan) | AB850653.1 | (18) |
| 103 | **HN7** | recombined | NPC | Asia (China, Hunan) | AB850655.1 | (18) |
| 104 | **HN8** | recombined | NPC | Asia (China, Hunan) | AB850657.1 | (18) |
| 105 | **HN9** | recombined | NPC | Asia (China, Hunan) | AB850659.1 | (18) |
| 106 | **HN10** | recombined | NPC | Asia (China, Hunan) | AB850645.1 | (18) |
| 107 | **HN11** | recombined | NPC | Asia (China, Hunan) | AB850646.1 | (18) |
| 108 | **HN12** | recombined | NPC | Asia (China, Hunan) | AB850647.1 | (18) |
| 109 | **HN13** | recombined | NPC | Asia (China, Hunan) | AB850650.1 | (18) |
| 110 | **HN14** | recombined | NPC | Asia (China, Hunan) | AB850651.1 | (18) |
| 111 | **HN15** | 1 | NPC | Asia (China, Hunan) | AB850654.1 | (18) |
| 112 | **HN16** | recombined | NPC | Asia (China, Hunan) | AB850656.1 | (18) |
| 113 | **HN17** | recombined | NPC | Asia (China, Hunan) | AB850658.1 | (18) |
| 114 | **HN18** | recombined | NPC | Asia (China, Hunan) | AB850660.1 | (18) |
| 115 | **HNNPC7** | 1 | NPC | Asia (China, Hunan) | LC150742.1 | (19) |
| 116 | **Saliva1** | 1 | Healthy Saliva | Europe (UK) | LN824142.1 | (3) |
| 117 | **NA19114** | 1 | PBL | Africa (Nigeria) | * | (20) |
| 118 | **NA19315** | 1 | PBL | Africa (Kenya) | * | (20) |
| 119 | **NA19384** | 1 | PBL | Africa (Kenya) | * | (20) |
| 120 | **K4123-Mi** | 1 | sLCL PBL | North America (USA) | KC440852.1 | (21) |
| 121 | **K4413-Mi** | 1 | sLCL PBL | North America (USA) | KC440852.1 | (21) |
| 122 | **AFB1b** | 2 | LCL | Unknown | LN827554.1 | (3) |
| 123 | **HKN14** | 1 | sLCL | Asia (China, Hong Kong) | LN824209.1 | (3) |
| 124 | **HKN15** | 1 | sLCL | Asia (China, Hong Kong) | LN827547.1 | (3) |
| 125 | **HKN19** | 1 | sLCL | Asia (China, Hong Kong) | LN824224.1 | (3) |
| 126 | **LCL_B958_delEber2** | 1 | LCL | North America (USA) | LN827739.1 | (3) |
| 127 | **pLCL-TRL1-post** | 1 | sLCL, PTLD | North America (USA) | LN824206.1 | (3) |
| 128 | **pLCL-TRL1-pre** | 1 | sLCL, PTLD | North America (USA) | LN824207.1 | (3) |
| 129 | **pLCL-TRL595** | 1 | sLCL, PTLD | North America (USA) | LN827559.1 | (3) |
| 130 | **sLCL-1.02** | 1 | sLCL | Africa (Kenya) | LN827558.1 | (3) |
| 131 | **sLCL-1.04** | 1 | sLCL | Africa (Kenya) | LN827585.1 | (3) |
| 132 | **sLCL-1.05** | 1 | sLCL | Africa (Kenya) | LN827581.1 | (3) |
| 133 | **sLCL-1.06** | 1 | sLCL | Africa (Kenya) | LN827566.1 | (3) |
| 134 | **sLCL-1.07** | 1 | sLCL | Africa (Kenya) | LN827565.1 | (3) |
| 135 | **sLCL-1.08** | 1 | sLCL | Africa (Kenya) | LN827552.1 | (3) |
| 136 | **sLCL-1.09** | 1 | sLCL | Africa (Kenya) | LN827574.1 | (3) |
| 137 | **sLCL-1.10** | 1 | sLCL | Africa (Kenya) | LN827573.1 | (3) |
| 138 | **sLCL-1.11** | 1 | sLCL | Africa (Kenya) | LN827550.1 | (3) |
| 139 | **sLCL-1.12** | 1 | sLCL | Africa (Kenya) | LN824205.1 | (3) |
| 140 | **sLCL-1.13** | 1 | sLCL | Africa (Kenya) | LN827579.1 | (3) |
| 141 | **sLCL-1.17** | 1 | sLCL | Africa (Kenya) | LN827577.1 | (3) |
| 142 | **sLCL-1.18** | 2 | sLCL | Africa (Kenya) | LN827563.2 | (3) |
| 143 | **sLCL-1.19** | 1 | sLCL | Africa (Kenya) | LN827562.1 | (3) |
| 144 | **sLCL-1.24** | 1 | sLCL | Africa (Kenya) | LN827568.1 | (3) |
| 145 | **sLCL-2.14** | 2 | sLCL | Africa (Kenya) | LN827560.1 | (3) |
| 146 | **sLCL-2.15** | 2 | sLCL | Africa (Kenya) | LN827591.1 | (3) |
| 147 | **sLCL-2.16** | 2 | sLCL | Africa (Kenya) | LN827580.1 | (3) |
| 148 | **sLCL-2.21** | 2 | sLCL | Africa (Kenya) | LN827587.1 | (3) |
| 149 | **sLCL-2.22** | 2 | sLCL | Africa (Kenya) | LN831023.1 | (3) |
| 150 | **sLCL-BL1.03** | 1 | sLCL | Africa (Kenya) | LN827582.1 | (3) |
| 151 | **sLCL-BL1.20** | 1 | sLCL | Africa (Kenya) | LN827571.1 | (3) |
| 152 | **sLCL-IS1.01** | 1 | sLCL, PTLD | Australia | LN827570.1 | (3) |
| 153 | **sLCL-IS1.03** | 1 | sLCL, PTLD | Australia | LN827595.1 | (3) |
| 154 | **sLCL-IS1.04** | 1 | sLCL, PTLD | Australia | LN827597.1 | (3) |
| 155 | **sLCL-IS1.06** | 1 | sLCL, PTLD | Australia | LN827584.1 | (3) |
| 156 | **sLCL-IS1.07** | 1 | sLCL, PTLD | Australia | LN827594.1 | (3) |
| 157 | **sLCL-IS1.08** | 1 | sLCL, PTLD | Australia | LN827553.1 | (3) |
| 158 | **sLCL-IS1.10** | 1 | sLCL, PTLD | Australia | LN827592.1 | (3) |
| 159 | **sLCL-IS1.11** | 1 | sLCL, PTLD | Australia | LN827569.1 | (3) |
| 160 | **sLCL-IS1.12** | 1 | sLCL, PTLD | Australia | LN827593.1 | (3) |
| 161 | **sLCL-IS1.13** | 1 | sLCL, PTLD | Australia | LN827578.1 | (3) |
| 162 | **sLCL-IS1.14** | 1 | sLCL, PTLD | Australia | LN827575.1 | (3) |
| 163 | **sLCL-IS1.15** | 1 | sLCL, PTLD | Australia | LN827586.1 | (3) |
| 164 | **sLCL-IS1.18** | 1 | sLCL, PTLD | Australia | LN827572.1 | (3) |
| 165 | **sLCL-IS1.19** | 1 | sLCL, PTLD | Australia | LN827588.1 | (3) |
| 166 | **sLCL-IS1.20** | 1 | sLCL, PTLD | Australia | LN827576.1 | (3) |
| 167 | **sLCL-IS2.01** | 2 | sLCL, PTLD | Australia | LN827589.1 | (3) |
| 168 | **X50-7** | 1 | LCL | North America (USA) | LN827555.1 | (3) |

BL, Burkitt lymphoma; GC, gastric carcinoma; HL, Hodgkin lymphoma; IM, infectious mononucleosis; LC, lung carcinoma; LCL, lymphoblastoid cell line; sLCL, spontaneous lymphoblastoid cell line; NPC, nasopharyngeal carcinoma; PTLD, post-transplant lymphoproliferative disease; *, Supplementary figure S9 of indicated reference

**References**

1. Dolan A, Addison C, Gatherer D, Davison AJ, McGeoch DJ. The genome of Epstein-Barr virus type 2 strain AG876. Virology. 2006;350(1):164-70.

2. Lin Z, Wang X, Strong MJ, Concha M, Baddoo M, Xu G, et al. Whole-genome sequencing of the Akata and Mutu Epstein-Barr virus strains. Journal of virology. 2013;87(2):1172-82.

3. Palser AL, Grayson NE, White RE, Corton C, Correia S, Ba Abdullah MM, et al. Genome diversity of Epstein-Barr virus from multiple tumor types and normal infection. Journal of virology. 2015;89(10):5222-37.

4. Lei H, Li T, Li B, Tsai S, Biggar RJ, Nkrumah F, et al. Epstein-Barr virus from Burkitt Lymphoma biopsies from Africa and South America share novel LMP-1 promoter and gene variations. Sci Rep. 2015;5:16706.

5. Liu Y, Yang W, Pan Y, Ji J, Lu Z, Ke Y. Genome-wide analysis of Epstein-Barr virus (EBV) isolated from EBV-associated gastric carcinoma (EBVaGC). Oncotarget. 2016;7(4):4903-14.

6. Chen JN, Zhou L, Qiu XM, Yang RH, Liang J, Pan YH, et al. Determination and genome-wide analysis of Epstein-Barr virus (EBV) sequences in EBV-associated gastric carcinoma from Guangdong, an endemic area of nasopharyngeal carcinoma. J Med Microbiol. 2018;67(11):1614-27.

7. Song KA, Yang SD, Hwang J, Kim JI, Kang MS. The full-length DNA sequence of Epstein Barr virus from a human gastric carcinoma cell line, SNU-719. Virus Genes. 2015;51(3):329-37.

8. Borozan I, Zapatka M, Frappier L, Ferretti V. Analysis of Epstein-Barr Virus Genomes and Expression Profiles in Gastric Adenocarcinoma. Journal of virology. 2018;92(2).

9. Baer R, Bankier AT, Biggin MD, Deininger PL, Farrell PJ, Gibson TJ, et al. DNA sequence and expression of the B95-8 Epstein-Barr virus genome. Nature. 1984;310(5974):207-11.

10. Weiss ER, Lamers SL, Henderson JL, Melnikov A, Somasundaran M, Garber M, et al. Early Epstein-Barr Virus Genomic Diversity and Convergence toward the B95.8 Genome in Primary Infection. Journal of virology. 2018;92(2).

11. Wang S, Xiong H, Yan S, Wu N, Lu Z. Identification and Characterization of Epstein-Barr Virus Genomes in Lung Carcinoma Biopsy Samples by Next-Generation Sequencing Technology. Sci Rep. 2016;6:26156.

12. Zeng MS, Li DJ, Liu QL, Song LB, Li MZ, Zhang RH, et al. Genomic sequence analysis of Epstein-Barr virus strain GD1 from a nasopharyngeal carcinoma patient. Journal of virology. 2005;79(24):15323-30.

13. Liu P, Fang X, Feng Z, Guo YM, Peng RJ, Liu T, et al. Direct sequencing and characterization of a clinical isolate of Epstein-Barr virus from nasopharyngeal carcinoma tissue by using next-generation sequencing technology. Journal of virology. 2011;85(21):11291-9.

14. Tso KK, Yip KY, Mak CK, Chung GT, Lee SD, Cheung ST, et al. Complete genomic sequence of Epstein-Barr virus in nasopharyngeal carcinoma cell line C666-1. Infect Agent Cancer. 2013;8(1):29.

15. Kwok H, Tong AH, Lin CH, Lok S, Farrell PJ, Kwong DL, et al. Genomic sequencing and comparative analysis of Epstein-Barr virus genome isolated from primary nasopharyngeal carcinoma biopsy. PloS one. 2012;7(5):e36939.

16. Kwok H, Wu CW, Palser AL, Kellam P, Sham PC, Kwong DL, et al. Genomic diversity of Epstein-Barr virus genomes isolated from primary nasopharyngeal carcinoma biopsy samples. Journal of virology. 2014;88(18):10662-72.

17. Tsai MH, Raykova A, Klinke O, Bernhardt K, Gartner K, Leung CS, et al. Spontaneous lytic replication and epitheliotropism define an Epstein-Barr virus strain found in carcinomas. Cell reports. 2013;5(2):458-70.

18. Tu C, Zeng Z, Qi P, Li X, Yu Z, Guo C, et al. Genome-Wide Analysis of 18 Epstein-Barr Viruses Isolated from Primary Nasopharyngeal Carcinoma Biopsy Specimens. Journal of virology. 2017;91(17).

19. Tu C, Zeng Z, Qi P, Li X, Guo C, Xiong F, et al. Identification of genomic alterations in nasopharyngeal carcinoma and nasopharyngeal carcinoma-derived Epstein-Barr virus by whole-genome sequencing. Carcinogenesis. 2018;39(12):1517-28.

20. Santpere G, Darre F, Blanco S, Alcami A, Villoslada P, Mar Alba M, et al. Genome-wide analysis of wild-type Epstein-Barr virus genomes derived from healthy individuals of the 1,000 Genomes Project. Genome Biol Evol. 2014;6(4):846-60.

21. Lei H, Li T, Hung GC, Li B, Tsai S, Lo SC. Identification and characterization of EBV genomes in spontaneously immortalized human peripheral blood B lymphocytes by NGS technology. BMC Genomics. 2013;14:804.
