# Supplementary material for: Immunoinformatic Analysis Reveals Antigenic Heterogeneity of Epstein-Barr Virus Is Immune-Driven
Source: Front Immunol. 2021 Dec 16;12:796379. doi: 10.3389/fimmu.2021.796379 (PMC8716887; doi:10.3389/fimmu.2021.796379)
Supplement: Supplementary file 3 [file Table_2.docx]

**Table S2: Number of CD4+ and CD8+ T-cell epitopes in EBV latent and lytic cycle antigens**

| **EBV antigen** | **Number of CD4+ T-cell epitopes** | **Number of CD8+ T-cell epitopes** |
| --- | --- | --- |
| **Latent cycle antigens** | | |
| EBNA1 | 33 | 10 |
| EBNA2 | 10 | 8 |
| EBNA3A | 5 | 14 |
| EBNA3B | 5 | 8 |
| EBNA3C | 14 | 11 |
| EBLA-LP | 1 | 1 |
| LMP1 | 9 | 8 |
| LMP2 | 9 | 26 |
| **Lytic cycle antigens** | | |
| BALF2 | 0 | 1 |
| BALF4 | 2 | 4 |
| BARF0 | 0 | 1 |
| BaRF1 | 1 | 5 |
| BARF1 | 0 | 4 |
| BcLF1 | 1 | 0 |
| BCRF1 | 0 | 1 |
| BFRF1 | 1 | 1 |
| BFRF3 | 0 | 1 |
| BHRF1 | 4 | 3 |
| BILF2 | 0 | 1 |
| BKRF2 | 1 | 1 |
| BLLF1 | 6 | 1 |
| BLLF3 | 0 | 2 |
| BMLF1 | 1 | 6 |
| BMRF1 | 2 | 7 |
| BNLF2a | 0 | 1 |
| BNLF2b | 0 | 1 |
| BNRF1 | 4 | 9 |
| BRLF1 | 4 | 15 |
| BVRF2 | 0 | 3 |
| BXLF2 | 1 | 4 |
| BZLF1 | 5 | 27 |
| BZLF2 | 1 | 0 |
|  | | |
| **Latent cycle antigens** | 86 | 86 |
| **Lytic cycle antigens** | 34 | 99 |
| **Total** | **120** | **185** |
