# Supplementary material for: Immunoinformatic Analysis Reveals Antigenic Heterogeneity of Epstein-Barr Virus Is Immune-Driven
Source: Front Immunol. 2021 Dec 16;12:796379. doi: 10.3389/fimmu.2021.796379 (PMC8716887; doi:10.3389/fimmu.2021.796379)
Supplement: Supplementary file 5 [file Table_4.docx]

| **Antigen** | **Position in B95.8 protein** | **HLA restriction**‡ | **B95.8 epitope sequence** | **Number of strains analyzed** | **% of strains with conserved epitope sequence†** | **Reference** |
| --- | --- | --- | --- | --- | --- | --- |
| **latent phase proteins** | | | | | | |
| EBNA1 | 607-619 | DQ2 | DGVDL-**PPWFPPMVEGAAA**-EGDDG | 150 | 100% | (Voo, Peng et al. 2005) |
| EBNA-LP | 41-55 | Multiple, DPB1*13:01 | PTRGG-**QEPRRVRRRVLVQQE**-EEVVS | 91 | 100% | (Brooks, Long et al. 2016)  Mautner et al., unpublished |
| **immediate early proteins** | | | | | | |
| BZLF1 | 11-25 | DRB3*01 | STSED-**VKFTPDPYQVPFVQA**-FDQAT | 157 | 100% | (Long, Leese et al. 2011) |
| BRLF1 | 139-153 | n.d. | ATIGT-**AMYKLLKHSRVRAYT**-YSKVL | 156 | 100% | Mautner et al., unpublished |
|  | 227-236 | DRB1*13:01 | KDVRA-**LIKTLPRASY**-SSHAG | 156 | 98,7% | Mautner et al., unpublished |
|  | 407-421 | DRB1*08 | KPTFL-**PPVKRKKGLRDSREG**-MFLPK | 156 | 98,7% | (Long, Leese et al. 2011) |
| **early proteins** | | | | | | |
| BaRF1 | 185-199 | DR7 | ANNYI-**SRDELLHTRAASLLY**-NSMTA | 157 | 100% | (Long et al., 2013) |
| BHRF1 | 45-57 | DR4 | LSPED-**TVVLRYHVLLEEI**-IERNS | 157 | 97,5% | (Schmidt and Misko 1995) |
|  | 171-189 | DR2 | WTLFL-**AGLTLSLLVICSYLFISRG**-RH | 157 | 98,0% | (White, Cross et al. 1996) |
| BMRF1 | 126-140 | DRB1*03:01 | SRPEF-**VKLTMEYDDKVSKSH**-HTCAL | 153 | 99,3% | (Long, Leese et al. 2011) |
|  | 370-384 | DRB5*01:02 | SNTAL-**ERPLAVQLARKRTSS**-EARQK | 154 | 100% | (Adhikary et al., 2007) |
| BFRF1 | 125-139 | DR7 | KNSII-**MLGQDDFIKFKSPLV**-FPAEL | 156 | 100% | (Long, Chagoury et al. 2013) |
| **late proteins** | | | | | | |
| BALF4 | 482-496 | DRB1*13:01 | GDLAR-**AWCLEQKRQNMVLRE**-LTKIN | 151 | 100% | (Adhikary, Behrends et al. 2006) |
|  | 575-589 | DRB1*08:01 | GQLGT-**DNEIFLTKKMTEVCQ**-ATSQY | 151 | 100% | (Adhikary, Behrends et al. 2006) |
| BcLF1 | 62-72 | DPB1*04:01 | GVYTN-**AIQYVRFLETA**-LAVSC | 150 | 100% | (Gary et al., 2018) |
| BLLF1 | 130-144 | DQB1*04:02 | LESVD-**VYFQDVFGTMWCHHA**-EMQNP | 150 | 99,3% | (Adhikary, Behrends et al. 2006) |
|  | 163-183 | DRw11 | PYIKW-**DNCNSTNITAVVRAQGLDVTL**-PLSLP | 150 | 98,0% | (Wallace, Wright et al. 1991) |
|  | 169-183 | DRB4*01:03 | NCNST-**NITAVVRAQGLDVTL**-PLSLP | 150 | 99,3% | Mautner et al., unpublished |
| BNRF1 | 915-929 | DRB1*15:02, DRB5*01:02 | VASDY-**LPEMFAEHPGLVFEV**-EERSV | 150 | 100% | Mautner et al., unpublished |
|  | 1006-1017 | DRB3*02:02 | NRSMH-**VSDYGYNEALAV**-SPLTG | 150 | 100% | Gary et al., 2018 |
|  | 1238-1252 | DRB1*15:01, DRB5*01:01 | FHSNG-**TDAWRFAMNYPRNPT**-EQGNI | 150 | 100% | (Milosevic, Behrends et al. 2006) |
| BXLF2 | 126-145 | DRB5*01 | MLSAP-**LEKQLFYYIGTMLPNTRPHS**-YVFYQ | 158 | 100% | (Long, Leese et al. 2011) |
| BZLF2 | 186-205 | DRB1*16 | TFKVY-**QIFGSHCTYVSKFSTVPVSH**-HECSF | 157 | 100% | (Long, Leese et al. 2011) |

**Table S4: CD4+ T-cell epitopes conserved in > 95% of all strains**

n.d. not determined, †considering polymorphisms in epitope and/ or FR; ‡more than one HLA molecule listed if restriction element not precisely defined or epitope presented on different HLA molecules

**Table S4: CD8+ T-cell epitopes conserved in > 95% of all strains analyzed**

| **Antigen** | **Position in B95.8 protein** | **HLA restriction**‡ | **B95.8 epitope sequence** | **Number of strains analyzed** | **% of strains with conserved epitope sequence†** | **Reference** |
| --- | --- | --- | --- | --- | --- | --- |
| **latent phase proteins** | | | | | | |
| EBNA1 | 508-517 | Cw03/04, A*68:02 | WVAGV-**FVYGGSKTSL**-YNLRR | 150 | 100% | (Ito, Demachi-Okamura et al. 2007, Fogg, Wirth et al. 2009) |
| EBNA3A | 596-604 | A2 | SPSQM-**SVRDRLARL**-RAEAQ | 150 | 100% | (Burrows, Gardner et al. 1994) |
| EBNA3B | 243-253 | B*27:02 | AFLMA-**RRARSLSAERY**-TLFFD | 156 | 100% | (Brooks, Colbert et al. 1998) |
| EBNA3C | 284-293 | A*02:01 | QTEEN-**LLDFVRFMGV**-MSSCN | 150 | 99,3% | (Kerr, Kienzle et al. 1996) |
|  | 285-293 | B37 | TEENL-**LDFVRFMGV**-MSSCN | 150 | 99,3% | (Shi, Smith et al. 1997) |
|  | 881-889 | B7 | SWSSP-**QPRAPIRPI**-PTRFP | 147 | 98,0% | (Hill, Worth et al. 1995) |
| LMP1 | 159-167 | A2 | LIIAL-**YLQQNWWTL**-LVDLL | 154 | 95,5% | (Khanna, Burrows et al. 1998) |
|  | 375-386 | B51.01 | GGDDD-**DPHGPVQLSYYD**- | 148 | 96,6% | (Meij, Leen et al. 2002) |
| LMP2 | 1-9 | B*35:01 | -**MGSLEMVPM**-GAGPP | 155 | 97,4% | (Straathof, Leen et al. 2005) |
|  | 121-134 | n.d. | GRGSM-**NPVCLPVIVAPYLF**-WLAAI | 154 | 99,4% | (Meij, Leen et al. 2002) |
|  | 125-133 | B53 | MNPVC-**LPVIVAPYL**-FWLAA | 154 | 99,4% | (Straathof, Leen et al. 2005) |
|  | 131-139 | A23 | PVIVA-**PYLFWLAAI**-AASCF | 154 | 99,4% | (Khanna, Burrows et al. 1996) |
|  | 264-272 | A2 | VDAVL-**QLSPLLGAV**-TVVSM | 154 | 100% | (Wang, Yao et al. 2009) |
|  | 293-301 | A2 | LSSPG-**GLGTLGAAL**-LTLAA | 154 | 99,4% | (Meij, Leen et al. 2002) |
|  | 329-337 | A*02:01 | MFLLM-**LLWTLVVLL**-ICSSC | 152 | 100% | (Lee, Thomas et al. 1996) |
|  | 356-364 | A2 | LLARL-**FLYALALLL**-LASAL | 152 | 99,3% | (Lautscham, Haigh et al. 2003) |
|  | 453-461 | A*02:06 | LSAWI-**LTAGFLIFL**-IGFAL | 151 | 96,0% | (Lee, Thomas et al. 1996) |
| **immediate early proteins** | | | | | | |
| BRLF1 | 25-38 | B18 | KQLGS-**LVSDYCNVLNKEFT**-AGSVE | 156 | 100% | (Pepperl, Benninger-Doring et al. 1998) |
|  | 25-33 | A*02:05 | KQLGS-**LVSDYCNVL**-NKEFT | 156 | 100% | (Hislop, Taylor et al. 2007) |
|  | 28-37 | A24 | GSLVS-**DYCNVLNKEF**-TAGSV | 156 | 100% | (Pepperl, Benninger-Doring et al. 1998) |
|  | 61-75 | n.d. | INEAK-**AHGREWGGLMATLNI**-CNFWA | 156 | 100% | (Abbott, Quinn et al. 2013) |
|  | 91-99 | B45 | RVRRR-**AENAGNDAC**-SIACP | 156 | 100% | (Pudney, Leese et al. 2005) |
|  | 101-115 | A24, C2 | NDACS-**IACPIVMRYVLDHLI**-VVTDR | 156 | 100% | (Pudney, Leese et al. 2005) |
|  | 109-117 | A*02:01 | PIVMR-**YVLDHLIVV**-TDRFF | 156 | 100% | (Saulquin, Ibisch et al. 2000) |
|  | 121-135 | n.d. | VVTDR-**FFIQAPSNRVMIPAT**-IGTAM | 156 | 100% | (Pepperl, Benninger-Doring et al. 1998) |
|  | 134-142 | A11 | RVMIP-**ATIGTAMYK**-LLKHS | 156 | 100% | (Pepperl, Benninger-Doring et al. 1998) |
|  | 145-159 | A3 | MYKLL-**KHSRVRAYTYSKVLG**-VDRAA | 156 | 100% | (Pepperl, Benninger-Doring et al. 1998) |
|  | 225-239 | A2 | DVKDV-**RALIKTLPRASYSSH**-AGQRS | 156 | 98,7% | (Pepperl, Benninger-Doring et al. 1998) |
|  | 393-407 | Cw4 | HQESD-**ERPIFPHPSKPTFLP**-PVKRK | 156 | 98,7% | (Pepperl, Benninger-Doring et al. 1998) |
|  | 441-455 | n.d. | VFEGR-**EVCQPKRIRPFHPPG**-SPWAN | 156 | 98,1% | (Pepperl, Benninger-Doring et al. 1998) |
|  | 592-605 | n.d. | LLHAM-**HISTGLSIFDTSLF**- | 156 | 100% | (Abbott, Quinn et al. 2013) |
| BZLF1 | 14-23 | B51.1 | EDVKF-**TPDPYQVPFV**-QAFDQ | 157 | 100% | (Rist, Neller et al. 2015) |
|  | 16-26 | B7 | VKFTP-**DPYQVPFVQAF**-DQATR | 157 | 100% | (Quinn, Zuo et al. 2014) |
|  | 18-26 | Cw3 | FTPDP-**YQVPFVQAF**-DQATR | 157 | 100% | (Rist, Neller et al. 2015) |
| **early proteins** | | | | | | |
| BALF2 | 418-426 | B27 | SPNLL-**ARYAYYLQF**-CQGQK | 153 | 100% | (Pudney, Leese et al. 2005) |
| BARF0 | 356-364 | A2 | ARDRA-**LLWAARPRL**-LLSLQ | 139 | 97,1% | (Kienzle, Sculley et al. 1998) |
| BaRF1 | 9-19 | n.d. | LLYVR-**DHEGFACLTVE**-THRNR | 157 | 98,7% | (Abbott, Quinn et al. 2013) |
|  | 53-63 | n.d. | EFYKF-**LFTFLAMAEKL**-VNFNI | 157 | 100% | (Abbott, Quinn et al. 2013) |
|  | 73-83 | n.d. | IDELV-**TSFESHDIDHY**-YTEQK | 157 | 100% | (Abbott, Quinn et al. 2013) |
|  | 151-159 | A*02:01 | KILVF-**LLIEGIFFI**-SSFYS | 157 | 100% | (Abbott, Quinn et al. 2013) |
|  | 208-217 | B*07:02 | TAKAD-**RPRATWIQEL**-FRTAV | 157 | 99,4% | (Abbott, Quinn et al. 2013) |
| BARF1 | 3-11 | A2 | MA-**RFIAQLLLL**-ASCVA | 160 | 99,4% | (Martorelli, Houali et al. 2008) |
|  | 50-58 | A2 | EVSWF-**KLGPGEEQV**-LIGRM | 160 | 99,4% | (Martorelli, Houali et al. 2008) |
| BFRF1 | 121-131 | n.d. | VDLPK-**NSIIMLGQDDF**-IKFKS | 156 | 100% | (Abbott, Quinn et al. 2013) |
| BHRF1 | 17-25 | B39 | LCIRD-**SRVHGNGTL**-HPVLE | 157 | 98,7% | (Brooks, Long et al. 2016) |
|  | 63-71 | A68 | IERNS-**ETFTETWNR**-FITHT | 157 | 98,1% | (Brooks, Long et al. 2016) |
|  | 171-185 | n.d. | WTLFL-**AGLTLSLLVICSYLF**-ISRGR | 155 | 99,4% | (Abbott, Quinn et al. 2013) |
| BLLF3 | 169-183 | n.d. | HRPTI-**FGRSGLAMQGILVKP**-CRWRR | 154 | 98,7% | (Abbott, Quinn et al. 2013) |
| BMLF1 | 205-219 | n.d. | DPFLQ-**SMLAVAAHPEIGAWQ**-KVQPR | 152 | 100% | (Abbott, Quinn et al. 2013) |
|  | 244-252 | n.d. | TKSTN-**KDTWLDARM**-QAIQN | 152 | 100% | (Steven, Annels et al. 1997) |
|  | 259-267 | A*02:01 | AIQNA-**GLCTLVAML**-EETIF | 152 | 100% | (Scotet, David-Ameline et al. 1996, Steven, Annels et al. 1997) |
|  | 330-344 | n.d. | KQLFY-**ITCATARQNKVVETL**-SSSYV | 152 | 100% | (Abbott, Quinn et al. 2013) |
|  | 376-384 | B18 | CRRRH-**DEVEFLGHY**-IKNYN | 152 | 99,3% | (Steven, Annels et al. 1997) |
| BMRF1 | 6-20 | n.d. | METTQ-**TLRFKTKALAVLSKC**-YDHAQ | 154 | 100% | (Abbott, Quinn et al. 2013) |
|  | 20-28 | A24 | AVLSK-**CYDHAQTHL**-KGGVL | 153 | 100% | (Pudney, Leese et al. 2005) |
|  | 86-100 | C3, C10 | PAAVS-**FRNLAYGRTCVLGKE**-LFGSA | 153 | 99,3% | (Steven, Annels et al. 1997) |
|  | 116-128 | B7 | LQFYK-**RPQGGSRPEFVKL**-TMEYD | 153 | 99,3% | (Pudney, Leese et al. 2005) |
|  | 208-216 | A*02:01 | GEACL-**TLDYKPLSV**-GPYEA | 153 | 98,7% | (Hislop, Annels et al. 2002) |
|  | 268-276 | B39, C6 | PILRF-**YRSGIIAVV**-AGLLT | 153 | 95,4% | (Steven, Annels et al. 1997) |
|  | 286-295 | A11,  B35, B53 | TSAGD-**LPLDLSVILF**-NHASE | 153 | 98,7% | (Steven, Annels et al. 1997) |
| BNLF2a | 50-58 | A2 | LVVLC-**VLFGLLCLL**-LI | 166 | 98,8% | (Bell, Abbott et al. 2009) |
| **late proteins** | | | | | | |
| BALF4 | 106-114 | A2 | KIVTN-**ILIYNGWYA**-DSVTN | 151 | 100% | (Khanna and Burrows 2000) |
|  | 190-198 | B35 | TELYD-**APGWLIWTY**-RTRTT | 151 | 100% | (Khanna and Burrows 2000) |
|  | 276-284 | A2 | GERRA-**FLDKGTYTL**-SWKLE | 151 | 100% | (Pudney, Leese et al. 2005) |
|  | 545-552 | B35 | RKSMR-**VPGSETMCY**-SRPLV | 150 | 98,7% | (Khanna and Burrows 2000) |
| BCRF1 | 3-11 | B27 | ME-**RRLVVTLQC**-LVLLY | 155 | 96,8% | (Saulquin, Bodinier et al. 2001) |
| BFRF3 | 127-137 | B*07:02 | VAQSA-**TPSVSSSISSL**-RAATS | 154 | 96,8% | (Abbott, Quinn et al. 2013) |
| BILF2 | 240-248 | B27 | LAGWG-**RRRKGWIPL**- | 155 | 100% | (Pudney, Leese et al. 2005) |
| BKRF2 | 120-128 | B60 | YGSFS-**VEDLFGANL**-NRYAW | 164 | 100% | (Abbott, Quinn et al. 2013) |
| BLLF1 | 863-871 | A2 | NLSML-**VLQWASLAV**-LTLLL | 151 | 99,3% | (Khanna, Sherritt et al. 1999) |
| BNRF1 | 409-419 | n.d. | SGLTA-**PPCPYAESSWA**-QAAVQ | 150 | 98,0% | (Abbott, Quinn et al. 2013) |
|  | 433-447 | n.d. | ELFSA-**LYPAPCISGYARPPG**-PSAVI | 150 | 99,3% | (Abbott, Quinn et al. 2013) |
|  | 709-719 | B41 | TVITL-**GEQGYKVSLDL**-REGTR | 150 | 100% | (Abbott, Quinn et al. 2013) |
|  | 929-943 | n.d. | GLVFE-**VEERSVGEVLQTLRS**-MNMYP | 150 | 99,3% | (Abbott, Quinn et al. 2013) |
|  | 1233-1247 | n.d. | QIACH-**FHSNGTDAWRFAMNY**-PRNPT | 150 | 100% | (Abbott, Quinn et al. 2013) |
|  | 1247-1257 | B*07:02 | RFAMN-**YPRNPTEQGNI**-AGLCS | 150 | 100% | (Abbott, Quinn et al. 2013) |
|  | 1281-1289 | A*02:01 | LCTDF-**WQWEHIPPA**-FGHPT | 150 | 100% | (Abbott, Quinn et al. 2013) |
| BVRF2 | 153-163 | C3 | RRGTT-**AVYGTDLAWVL**-KHFSD | 151 | 100% | (Abbott, Quinn et al. 2013) |
|  | 257-271 | n.d. | PPAST-**DPATMLSGNAGEGAT**-ACGGS | 151 | 98,7% | (Abbott, Quinn et al. 2013) |
| BXLF2 | 46-60 | n.d. | TELMA-**KVPGLSPEALWREAN**-VTEDL | 158 | 100% | (Abbott, Quinn et al. 2013) |
|  | 225-233 | A*02:01 | QSGDY-**SLVIVTTFV**-HYANF | 155 | 98,7% | (Khanna, Sherritt et al. 1999) |
|  | 420-428 | A*02:01 | TVMRE-**TLFIGSHVV**-LRELR | 155 | 99,4% | (Khanna, Sherritt et al. 1999) |
|  | 542-550 | A*02:01 | RLDKV-**LMIIPLINV**-TFIIS | 155 | 100% | (Khanna, Sherritt et al. 1999) |

n.d. not determined; †considering polymorphisms in epitope and/ or FR; ‡more than one HLA molecule listed if restriction element not precisely defined or epitope presented on different HLA molecules
